# Druggists as Prescribers

**Published:** 1877-09

**Authors:** 

**Affiliations:** Uptonville


### Correspondence.

UPTONVILLE, Aug. 16, 1877.

EDITORS MEDICAL JOURNAL: Five years ago I received, at the hands of the President of one of the most honored Medical Schools in America, a diploma conferring upon me the degree of M. D., with all the rights and privileges thereunto pertaining.

Four years of pupilage, after a careful preparatory course of classical and scientific study, were passed under the direction of one of the most eminent physicians in New York State. Under his care and subsequently as hospital interne, I derived as complete practical and theoretical knowledge of medicine and surgery as diligent study and close observation could give me, and without being inclined to be at all egotistical, but simply for the purpose of illustrating what I have to say, I graduated with better preparation for the practice of medicine than do four-fifths of the yearly created doctors turned out by our numerous medical schools.

Spending but a short time in recreation, I soon settled upon the town of Uptonville as the seat of my future labors in behalf of suffering humanity. As Uptonville is a fictitious name, I will briefly describe the place, not sufficiently, however, to enable any of your readers to recognize the locality. Situated at the junction of three large railway lines, it derives considerable importance from being a railway centre. The chief revenue of the inhabitants is, however, derived from its manufacturing interests, there being several large iron and hardware manufactories situated here, beside other and smaller factories. The population five years ago was something less than one hundred thousand; now it has considerably exceeded that number. The people are refined and intelligent, they maintain excellent schools and seminaries, take considerable interest in the arts and sciences, and in short are all that could be expected of the inhabitants of a place which has not yet marked four score upon its calender.

Renting and fitting up an office, in what I was informed was an excellent location. I placed my neat sign at the door and waited for business. My first calls were from the members of the medical profession residing in my near vicinity. Some kindly encouraged me, and others, doubtless, intending to be equally kind, spoke dolefully of the obstacles in the path of the young practitioner, and with, no doubt, perfectly disinterested (?) motives, told me that of all places, Uptonville was the worst to locate in. I was importuned to join various medical societies, and soon discovered that they were severally, with one or two exceptions, "run" by various factions. Selecting those which seemed most free from cliquism, I entered heartily into the objects of the associations, of which I became a member. My first quarter's rent came due in a very short time, and I found that of the fifty dollars then due, only fifteen had been realized from my practice. The location in which I had established myself was such that I could not depend upon the wealthier classes for practice. My income must be derived from what is termed the middle class, but I soon learned that there were many others equally anxious for the practice upon which I was to depend for my daily bread. The druggists at the corner above, upon my advent into their neighborhood had kindly called upon me, had held out the most flattering encouragement, kindly presented me with sample bottles of the most tempting elixers, pills and powders, and upon taking leave had expressed a desire to send me some prescription blanks, with the hope, that, as theirs was the only store in the vicinity, I would favor them with a share of my patronage. In due time the blanks came; they were printed in the usual form, and were quite convenient. Shortly after my interview with the representative of Messrs. Mortar & Pestle an incident occurred which showed how little they were inclined to reciprocate by giving me, or any other doctor in their vicinity a share of their patronage.

A lady called at my office, wishing me to prescribe for her little daughter who had, she thought a mild attack of intermittent fever. I declined to prescribe unless I could see the patient, and the mother left, saying she might perhaps bring her to my office the next day. A day or two subsequently the mother informed me, on meeting on the street, that she had called at the drug store on the corner, and that one of the proprietors had given her some "billious" pills, which would, he thought, be just what her daughter needed. I replied that as the druggist could diagnose and prescribe without seeing the patient, he had better put out a sign as Clairvoyant Physician. Surely "fools rush in where (doctors) fear to tread." I soon found that it was the invariable practice of nearly all druggists in Uptonville to prescribe for all the ills that flesh is heir to, arriving at a diagnosis with a facility that would put to shame the slow and antiquated methods of the most skilled hospital Physician.

When I inquired why people should have much faith in the skill of the druggist as a prescriber, I found thas there were two reasons:—first, it was cheaper to simply pay the druggist for the medicine than to pay the doctor for his advice and the druggist for compounding his prescription; second, the people had come to learn that the druggist knew what this or that doctor prescribed, and that he would give them Dr. A's iron mixture, Dr. B's nervous sedative, Dr. D's gargle, Dr. D's anti-acid mixture, and so on to the end of the list, without putting them to the trouble of calling upon their physician. Of course the question never for a moment arose, whether Dr. A, B, C, or D would prescribe just these remedies in this case, but that made no difference, as long as the druggist got his pay.

While making these investigations and some others, my second quarter passed, and while I am trying to make out how I can pay fifty dollars rent, to say nothing of board and other *incidental* expenses out of eighty dollars charged on my books and twenty in cash, collected, I will leave your readers to digest what I have already revealed, waiting until my next to detail my experience in the care of the pauper population who always flock to a "young doctor." Yours,

NIL DESPERANDUM.

